# Identification of a Novel Serological Marker in Seronegative Rheumatoid Arthritis Using the Peptide Library Approach

**DOI:** 10.3389/fimmu.2021.753400

**Published:** 2021-10-05

**Authors:** Caterina Bason, Alessandro Barbieri, Nicola Martinelli, Bianca Olivieri, Giuseppe Argentino, Elena Bartoloni, Ruggero Beri, Gnaneshwer Jadav, Antonio Puccetti, Elisa Tinazzi, Claudio Lunardi

**Affiliations:** ^1^ Department of Medicine, University of Verona, Verona, Italy; ^2^ Department of Laboratory Medicine, Boston Children’s Hospital, Harvard Medical School, Boston, MA, United States; ^3^ Division of Rheumatology, Department of Medicine, University of Perugia, Perugia, Italy; ^4^ Department of Experimental Medicine, Section of Histology, University of Genova, Genova, Italy

**Keywords:** peptide library approach, RA-peptide, seronegative rheumatoid arthritis, autoantibodies, autoantigen targets

## Abstract

Rheumatoid arthritis (RA) is a systemic autoimmune disease characterized by chronic inflammation mainly affecting the joints leading to cartilage and bone destruction. The definition of seropositive or seronegative RA is based on the presence or absence of rheumatoid factor (RF) and anti-citrullinated peptide antibodies (ACPAs). Other autoantibodies have been identified in the last decade such as antibodies directed against carbamylated antigens, peptidyl-arginine deiminase type 4 and v-Raf murine sarcoma viral oncogene homologue B. In order to identify relevant autoantigens, we screened a random peptide library (RPL) with pooled IgGs obtained from 50 patients with seronegative RA. Patients’ sera were then used in an ELISA test to identify the most frequently recognized peptide among those obtained by screening the RPL. Sera from age- and sex-matched healthy subjects were used as controls. We identified a specific peptide (RA-peptide) recognized by RA patients’ sera, but not by healthy subjects or by patients with other immune-mediated diseases. The majority of sera from seronegative and seropositive RA patients (73.8% and 63.6% respectively) contained IgG antibodies directed against the RA-peptide. Interestingly, this peptide shares homology with some self-antigens, such as Protein-tyrosine kinase 2 beta, B cell scaffold protein, Liprin-alfa1 and Cytotoxic T lymphocyte protein 4. Affinity purified anti-RA-peptide antibodies were able to cross react with these autoantigens. In conclusion, we identified a peptide that is recognized by seropositive and, most importantly, by seronegative RA patients’ sera, but not by healthy subjects, conferring to this epitope a high degree of specificity. This peptide shares also homology with other autoantigens which can be recognized by autoantibodies present in seronegative RA sera. These newly identified autoantibodies, although present also in a percentage of seropositive RA patients, may be considered as novel serum biomarkers for seronegative RA, which lacks the presence of RF and/or ACPAs.

## Introduction

Rheumatoid arthritis (RA) is an autoimmune disease characterized by chronic joint inflammation and synovial hyperplasia that lead to progressive and destructive arthritis. It is estimated that RA has a prevalence of 1% of world population (1) with a higher frequency in women and elderly people (2, 3).

Early diagnosis and treatment are essential in order to prevent disease progression and irreversible joint damage (4).

The diagnosis of RA is based on clinical features, radiographic images and serological markers, such as rheumatoid factor (RF) and anti-citrullinated peptide antibodies (ACPAs) (3). The presence of such autoantibodies in the serum of RA patients allows to distinguish seropositive RA from seronegative RA patients. It is estimated that one third of RA patients have no ACPAs.

These two forms of RA are characterized by different genetic and environmental risk factors, differences in preclinical and early phases, and different synovial fluid cytokine profile (2).

Seronegative RA patients have, indeed, a more active disease in the initial phase and have a slower response to treatment compared with seropositive RA patients (5). However, no differences in the treatment of the two subsets of the disease are provided by the current guidelines (6).

ACPAs are pathogenetic autoantibodies that are highly specific for RA (7). Anti-cyclic citrullinated peptide antibodies (anti-CCP2) are the most commonly ACPAs measured in patients’ serum. These antibodies are useful for distinguishing RA from other rheumatic diseases and they appear to be highly predictive of future RA development in either healthy subjects or in patients with undifferentiated arthritis (8, 9).

The presence of ACPAs is also related to the development of bone erosion (10, 11). Citrullinated peptides may be potential autoantigens and may be recognized by T cells that are activated and either contribute to the pro-inflammatory environment that may cause joint damage or trigger B cells to produce autoantibodies (12). Both ACPAs and inflammatory cytokines can be found even 2-4 years before the clinical outset of the disease (13, 14).

The use of the combined detection of RF and ACPAs makes the diagnosis of seropositive RA more accurate (15). On the contrary, in seronegative RA patients there is not a specific marker currently available; therefore, much effort should be made to identify markers present in this form of the disease.

Indeed, other autoantibodies associated with RA have also been identified, such as anti-carbamylated (anti-CarP), anti-v raf murine sarcoma viral oncogene homologue B1 (anti-BRAF) and anti-human peptidyl-arginine deiminase type 4 (anti-PAD4) antibodies (16). Anti-CarP antibodies are present in serum and synovial fluid (SF) of both seronegative and seropositive RA patients and are correlated with more severe disease and radiographic progression (17). In patients with arthralgias, the presence of anti-CarP antibodies predicts the development of RA independently of anti-CCP2 antibodies (18–21). Moreover, anti-CarP antibodies can be found in the sera of healthy first-degree relatives (HFDRs) of RA patients and their prevalence is significantly higher than in normal healthy subjects (22).

Anti-BRAF antibodies are detected in RA patients’ serum and are able to activate BRAF kinase, which is the first step in mitogen-activated protein kinase (MAP kinase) activation, leading to pro-inflammatory cytokine production and joint inflammation (23).

Anti-PAD4 antibodies are present in 36-42% of RA patients and appear to be specific markers for RA, as well as associated with more severe disease (24).

Recently, Colasanti et al., combining both proteomic and immunological approaches, were able to identify Hcy-A1AT (Homocysteinylated alpha 1 antitrypsin) as a new antigenic target in seronegative RA (25).

However, while there is an effective combination of serological markers in the diagnosis of seropositive RA, a specific marker is not currently available for the diagnosis of seronegative arthritis. Also, for this reason, diagnosis of seronegative RA is more difficult and may be delayed.

The aim of this work was to identify possible markers of seronegative RA. To this aim, we have used a random peptide library approach, that we have previously successfully applied to identify novel autoantigen targets in other autoimmune diseases (26–28).

## Material and Methods

### Study Population

In this study, 50 patients affected by seronegative RA (13 males and 37 females, mean age: 65.7 ± 10.83 years) and 25 by seropositive RA (7 males and 18 females, mean age: 55.88 ± 12.15 years) were enrolled by the Rheumatology Unit of the Perugia University Hospital. A cohort of 30 seronegative RA (8 males and 22 females, mean age: 62.57 ± 18.26) and 30 seropositive RA (5 males and 25 females, mean age: 58.9 ± 11.45 years) was enrolled by the Unit of Autoimmune Diseases at the University Hospital of Verona, as validation group.

The diagnosis of RA was performed according to the 2010 American College of Rheumatology/European League Against Rheumatism classification criteria for RA (3). All the subjects were screened for both RF and ACPAs, which were absent in seronegative RA patients.

A further group of 30 patients affected by other immune-mediated diseases, such as systemic sclerosis (SSc) (29), spondyloarthritis (SA) (30) and psoriatic arthritis (PsA) (31) was also included in the study. Finally, 25 age- and sex-matched healthy donors (HD) were included as a control group.

Blood was collected and centrifuged at 3000xg for 10 min. All sera were aliquoted and frozen at -20°C until used.

Written informed consent was obtained from adult patients and healthy donors. The study was approved by the local Ethical Committee of the Azienda Ospedaliera Universitaria Integrata of Verona, Verona, Italy (protocol number: 1538, version number 3) and by Comitato Universitario di Bioetica of Perugia, Italy (identification code: 2013-012 approved 4-4-2013).

### Peptide Library

The screening procedure of the FliTrx random dodecamer peptides library (FliTrx Panning Kit, Invitrogen, Carlsbad, CA, USA) has been described elsewhere by our group (26–28).

Briefly, a random peptides library was screened with pooled immunoglobulins (IgGs) obtained from the sera of 50 patients with seronegative RA, according to the manufacturer’s instructions. To remove the bacteria expressing peptides not related to the disease, a ‘pre-panning’ step with pooled IgGs obtained from 25 healthy donors was carried out,thereby allowing to discard non-specific peptides without the loss of disease-related data. Then we proceed with five sequential cycles of bio-panning. The enriched library was grown, and single colonies were selected, expanded and incubated with tryptophan to induce the expression of the fusion peptides. Bacteria were then lysed in sample buffer and tested by western blot procedures with the pooled IgG fraction from seronegative RA to identify positive clones. DNA was then extracted from these clones and sequenced. Peptide sequence was deduced from DNA sequence. A set of 17 out of 33 peptides was synthesized and used in an ELISA assay to test individual sera. The remaining 16 peptides were excluded due to a low amount of plasmid DNA isolation or because the DNA sequence did not yield a consensus peptide (e.g. DNA sequence contained stop codons).

### Peptide Synthesis

All the five synthetic peptides, including RA-peptide (LAVLANLASRTL), BANK-1 peptide (DMILANLSIKKK), PYK2/FADK2 peptide (AKVLANLAHPPA), LIPRIN-1 peptide (ASVLANVAQAFE) and CTLA-4 peptide (HKAQLNLATRTW) were purchased from TIBMolbiol (Genoa, Italy).

### Enzyme-Linked Immunosorbent Assays (ELISA)

The ELISA test for antibody binding to the synthetic peptides has already been described elsewhere (26–28, 32), and was carried out with minor modifications (see **Supplementary Material and Methods**).

All serum samples were diluted 1:100 in diluting buffer.

The absorbance value (O.D.) for each sample was calculated subtracting the O.D. plates with serum and no peptide to the O.D. plates with serum and peptide. O.D. values higher than the mean plus 3 standard deviations (S.D.) of O.D. value measured in control group were considered as positive (cutoff 0.079), as previously reported (32). The experiment has been carried out in duplicate (see **Supplementary Material and Methods**).

### Binding of Affinity Purified Abs to the Peptides

Affinity purified peptide Abs were obtained from sera of 15 seronegative RA patients (see **Supplementary Material and Methods**).

Different dilutions ranging from 20 µg/mL to 1.25 µg/mL of affinity purified Abs were tested in a plate coated with RA-peptide. As internal negative control, affinity purified anti-irrelevant-peptide Abs were used at the same dilutions.

The same procedure was also used to assess the direct binding of the other purified Abs (anti-Pyk/Fadk2, anti-Bank-1, anti-Liprin-1, anti-CTLA-4 and anti-irrelevant peptide) to their specific peptides (using plates coated with Pyk/Fadk2, Bank-1, Liprin-1 and CTLA-4, respectively) and to test the ability of the purified Abs to cross-recognize the other peptides, at the same dilution conditions.

For inhibition test, we pre-incubated the purified anti-RA-peptide Abs (20 µg/mL) with increasing concentration of competitors (synthetic RA-peptide or irrelevant peptide: 12.5, 25, 50, 100, 200 µg/mL) for 1 hour at 37 °C. The mixtures were then transferred to the RA-peptide coated plate. The assay was performed as the direct binding test above described. Results were expressed as inhibition percentage.

### Cell Culture

Normal Human Fibroblast-Like Synoviocytes (HFLS), Human Fibroblast-Like Synoviocytes: Rheumatoid Arthritis (HFLS-RA) and human synoviocytes complete growth medium were purchased from Cell applications (San Diego, CA, USA). Normal human dermal fibroblast (NHDF) and their growth media were purchased from Promocell Bioscience Alive (Heidelberg, Germany). Cells were cultured in standard conditions at 37°C in 5%CO_2_ and used between passages 3 and 6.

Human promyelocytic leukemia cell (HL-60) and Burkitt lymphoma cell (DAUDI) lines were purchased from ATCC (Manassas, VA, USA) and routinely maintained in RPMI-1640 medium (Sigma-Aldrich, St. Louis, MO), supplemented with 10% heat-inactivated fetal bovine serum (Sigma), 2 mM stable glutamine, 100U/ml penicillin and 100U/ml streptomycin (Sigma) in a humidified atmosphere with 5% CO_2_ at 37°C.

### Isolation of B and T Lymphocytes From Healthy Subjects

Normal B or T lymphocytes were isolated from PBMCs after blood separation on Ficoll HyPaque Plus (GE Healthcare, Little Chalfont, UK) and purification by negative selection with EasySep™ Human B Cell isolation Kit or EasySep™ Human T Cell isolation Kit (Stemcell Technologies, Vancouver, BC, Canada). Purity of B or T lymphocyte preparations were evaluated by flow cytometry with anti-CD19 mAb and with anti-CD3 mAb (BD Biosciences, San Jose, CA, USA).

### Immunoprecipitation

B, T and PBMCs or Synoviocytes (HFLS and HFLS-RA) were lysed in cold lysis buffer (0.5% Nonidet P-40, 10 mM TRIS [pH 7.4], 0.15 M sodium chloride, 5 mM magnesium chloride) and protease inhibitors (Sigma, Saint Louis, MO). Protein concentration of cell lysates was determined using the Pierce BCA Protein Assay Kit (Thermo Scientific, Rockford, IL). All cell lysates were immunoprecipitated with anti-RA specific peptide antibodies coupled to protein A magnetic beads using SureBeads™ Protein A Magnetic Beads (BioRad Laboratories, Inc, Hercules, CA, United States) following manufacturer’s instructions for standard immunoprecipitation protocol. Purified target proteins (20 µg) were run in a 10% SDS-PAGE gel and transferred to nitrocellulose membrane (Amersham Bioscience, Piscataway, New Jersey, United States). Immunoblots were performed using the following primary antibodies: anti-RA specific peptide antibodies, mouse polyclonal antibodies directed against PYK2 (Novus Biologicals, Littleton, CO, USA), rabbit polyclonal antibodies directed against Liprin (Novus Biologicals Littleton, CO, USA) and rabbit polyclonal antibodies directed against Bank (Abgent, San Diego, CA, USA) followed by their specific peroxidase-linked secondary antibodies. Blots were then developed using chemiluminescent substrate (Amersham; GE Healthcare Life Sciences, Milan, Italy) and images were acquired by Image Quant Las 4000mini (GE Healthcare Life Sciences, Milan, Italy).

### Statistical Analysis

All calculations were performed using the IBM SPSS 23.0 (IBM Inc., Armonk, NY) or GraphPad Prism 5 (GraphPad Software, San Diego, CA) statistical packages. Continuous variables are shown as mean values ± standard deviations. With the aim to compare O.D. values of patients with seronegative RA with those of i) patients with seropositive RA, ii) patients with immune-mediated diseases, or iii) healthy donors, a two-tailed non-parametric Mann-Whitney test was used. Analyses of receiver-operating characteristic (ROC) were carried out and ROC curves were plotted with area under the curve (AUC) as an indicator of the diagnostic value. Within the group of seronegative RA patients, more homogeneous and thus allowing the use of parametric tests, continuous variables having a skewed distribution (e.g. O.D., ESR, CRP) were ln-transformed and the statistical analyses were performed on ln-transformed values. However, for sake of clarity, also their results have been reported as mean values ± standard deviations. Differences of continuous variables between sample’s subgroups were analyzed by t-test, while correlations were assessed by Pearson’s analysis. Categorical variables are reported as relative proportions and were analyzed by chi-square test, with chi-square for linear trend when appropriate. A P value < 0.05 was considered as statistically significant.

## Results

### Clinical and Laboratory Features and Treatment of RA Patients

The main clinical features, biochemical characteristics and treatment of all seronegative RA patients included in the study are summarized in **Table 1**. **Supplementary Table 1** shows the data related to patients with seropositive RA, used as control. The RA patients were predominantly female and the gender percentage in the two seronegative RA groups were equally balanced.

**Table 1:** Clinical and laboratory features and treatment of seronegative rheumatoid arthritis (RA) patients from the Perugia (Pg) and Verona (Vr) cohorts.

	RA (Pg)	RA (Vr)
	(n=50)	(n=30)
*Clinical characteristics*		
**Age (years)**	65.7 ± 10.83	62.57 ± 18.26
**Gender: female/male**	37/13	22/8
**Age at diagnosis (years)**	57.32 ± 11.11	55.9 ± 17.45
**Disease duration (years)**	8.38 ± 5.51	6.67 ± 5.46
**Erosion**	5/44	6/19
**presence/absence, n.**	(n= 49)	(n= 25)
**Tender joint**	1.69 ± 2.52	1.6 ± 2.42
**count (0-10)**	(n= 49)	(n= 25)
**Swollen joint**	0.71 ± 1.84	1.56 ± 2.43
**count (0-10)**	(n= 49)	(n= 25)
**DAS28-ESR**	2.59 ± 1.10	2.76 ± 1.30
	(n=50)	(n= 30)
*Therapy*	**RA**	**RA**
	**(n=50)**	**(n=30)**
**Methotrexate (%)**	20 (40%)	12 (40%)
**Hydroxychloroquine (%)**	19 (38%)	9 (30%)
**Leflunomide (%)**	9 (18%)	5 (16.7%)
**Biological/others (%)**	2 (4%)	2 (6.7%)
**n° treatment (%)**	0 (0%)	2 (6.7%)
**Prednisolon/prednisone (%)**	16 (32%)	14 (56%)
	(n=50)	(n=25)
*Inflammatory markers*	**RA**	**RA**
**ESR (mm/h)**	24.66 ± 19.05	21.36 ± 14
	(n=50)	(n=28)
**CRP (mg/dl)**	0.70 ± 1.01	0.67 ± 0.92
	(n= 50)	(n=28)

RA, rheumatoid arthritis; DAS 28, Disease activity score 28; ESR, Erythrocyte sedimentation rate; CRP, C reactive protein. Values are shown as mean ± SD and as percentage (%).

### Use of Random Peptide Library and ELISA Test

A dodecamer random peptide library was used to identify possible relevant autoantigens in seronegative RA. An IgG pool derived from the first cohort of 50 patients with seronegative RA was used to screen the peptide library, while a pool of IgGs from 25 healthy donors was used for the pre-screening step (see material and methods). Finally, a set of 17 putative peptides was used to evaluate the binding of individual patient’s sera

**Figure 1:**
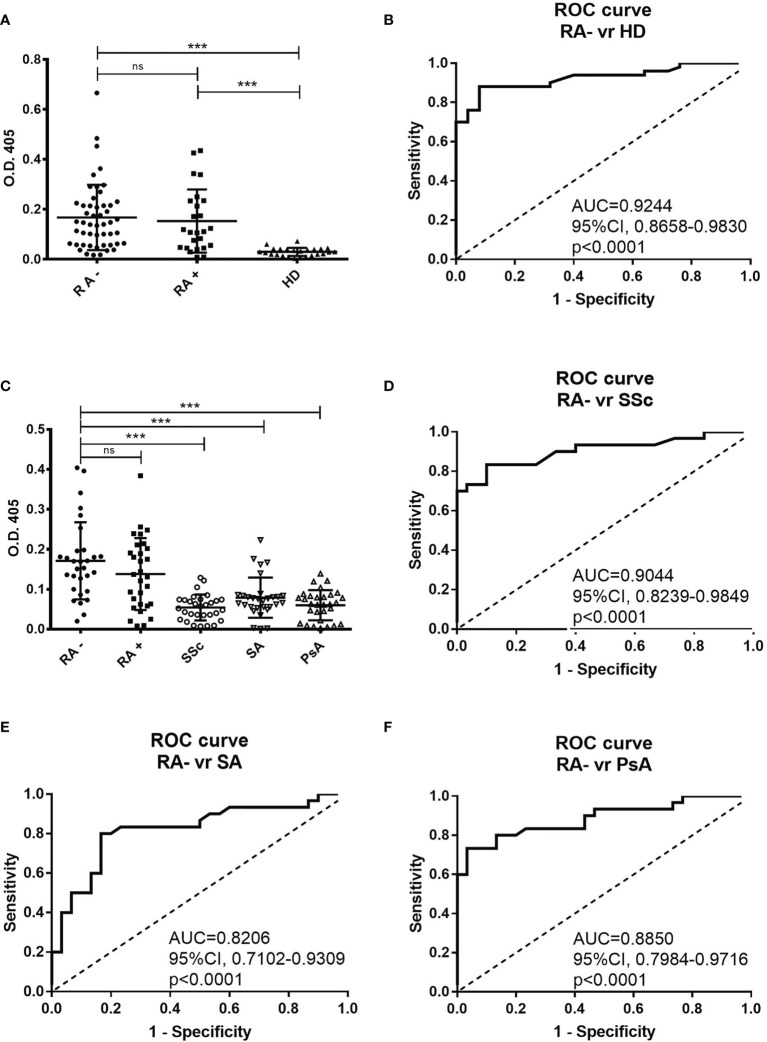
Direct binding of individual patients’ and healthy donors’ sera to RA-peptide obtained from peptide library screening. **(A)** RA**-**peptide is recognized by serum IgG antibodies of seronegative RA patients (●), by serum IgG of seropositive RA patients (■), but not by serum IgG of healthy donors (▲). The results are expressed as absorbance (O.D.) at 405 nm. Results shown are the mean values of three independent experiments. Bar line represents the mean absorbance value and standard deviation for each group. The differences among the different groups of patients and the healthy donors were determined by non-parametric Mann-Whitney test. Statistical significance is defined as following: ***P < 0,0001, ns, not significant. **(B)** Receiver operating characteristic (ROC) analysis of O.D. value of serum from seronegative RA patients and healthy donors. AUC= area under the curve. **(C)** Binding of RA-peptide by serum IgG antibodies of the validation cohort of seronegative RA (●), seropositive RA (■), and of patients with SSc (○), with SA (▽) and with PsA (△). The results are expressed as absorbance (O.D.) at 405 nm. Lines indicate the mean absorbance value and standard deviation for each group. The non-parametric Mann-Whitney test was used to compare the different groups. ***P < 0,0001, ns, not significant. **(D–F)** Receiver operating characteristic (ROC) analysis of O.D. value of serum from seronegative RA patients and SSc or SA or PsA patients, respectively. RA, Rheumatoid arthritis; SSc, Systemic sclerosis; SA, Spondyloarthritis; PsA, Psoriatic arthritis.

By using a direct ELISA test, we identified a peptide (RA-peptide: LAVLANLASRTL) that was recognized by individual serum IgG of 70% of patients with seronegative RA (35 out of 50; 9 men and 26 women) (**Figure 1A**). Such reactivity was not detected by the individual sera of 25 healthy subjects, using a O.D. cutoff of 0.079 (mean ± 3 s.d).

We also analyzed the sera of 25 seropositive RA patients and found that 64% of them recognized the RA-peptide (16 out of 25; 6 men and 10 women) (**Figure 1A**). Statistically significant differences were found in the binding to RA-peptide between the seronegative RA patients’ and healthy donors’ sera (absorbance mean ± s.d.: 0.1676 ± 0.1308 vs 0.02972 ± 0.01638, p<0.0001) or between the seropositive RA and healthy donors (absorbance mean ± s.d: 0.1532 ± 0.1264 vs 0.02972 ± 0.01638, p<0.0001), but not between seronegative and seropositive patients (p>0.05).

The potential diagnostic value of anti-RA-peptide antibodies in either seronegative or seropositive RA patients was assessed by a receiver operating characteristic (ROC) curve. The area below the ROC curve (AUC) was 0.9244 [95% confidence interval (CI) = 0.8658 to 0.9830, p<0.0001] between anti-peptide Abs of seronegative RA patients and healthy donors (**Figure 1B**) and 0.8840 [95% confidence interval (CI) =0.7797 to 0.9883, p<0.0001] between anti-peptide Abs of seropositive RA patients and healthy donors (**Supplementary Figure 1A**). No significant difference was found in ROC curve between the seronegative and seropositive RA (p=0.5514) (**Supplementary Figure 1B**).

In order to validate the binding specificity of RA-peptide, a second study sample of seronegative RA patients (n=30) was analyzed, as well as a group of patients with other immune-mediated diseases,like SA, PsA, and SSc (n=30).

In the validation analysis 83.3% of subjects with seronegative RA (25 out of 30; 7 men and 18 women) and 66.7% of patients with seropositive RA (20 out of 30; 5 men and 15 women) had serum IgG antibodies able to recognize the RA-peptide (**Figure 1C**).

Five out of 30 (16.7%) patients with SSc, 12 out of 30 (40%) with SA and 10 out of 30 (33.3%) with PsA showed a low reactivity to RA-peptide. Only a SA patient had a strong value of absorbance. There were differences in the absorbance between the seronegative RA and SSc patients (0.1710 ± 0.09651 vs 0.0543 ± 0.03228, p<0.0001) or SA patients (0.0788 ± 0.05045, p<0.0001) or PsA patients (0.05997 ± 0.03771, p<0.0001), but not between seronegative and seropositive patients (0.1710 ± 0.09651 vs 0.1380 ± 0.09002, p>0.05) (**Figure 1C**). Differences were also observed by comparing seropositive RA patients with SSc/SA/PsA patients (p=0.0001, p=0.0089, p=0.0004, respectively).

In the ROC curve analysis, AUC was 0.9044 [95% confidence interval (CI) = 0.8239 to 0.9849, p<0.0001] when anti-peptide Abs of seronegative RA and SSc subjects were compared (**Figure 1D**); 0.8206 [95% confidence interval (CI) = 0.7102 to 0.9309, p<0.0001] between RA and SA (**Figure 1E**); 0.8850 [95% confidence interval (CI) =0.7984 to 0.9716, p<0.0001] between RA and PsA (**Figure 1F**).

Similarly to the first cohort analysis, ROC curves between seropositive RA and seronegative RA were not significant (**Supplementary Figure 1C**), while the comparison of seropositive RA with SSc/SA/PsA showed significant differences in AUC (**Supplementary Figures 1D–F**).

Notably, many patients with SSc, SA and PsA showed a slight positivity of anti-RA-peptide Abs with a cutoff of 0.079. Therefore, on the basis of ROC curve analysis, we decided to increase the cutoff value to an arbitrary threshold of 0.090. Such threshold allows to preserve the sensibility of the biomarker (only 2 subjects became negative at testing with the new threshold in seropositive (n=1) and seronegative (n=1) RA cohorts), while the specificity was improved [3 out of 30 (10%) patients with SSc, 5 out of 30 (16.7%) with SA and 5 out of 30 (16.7%) with PsA showed a reactivity to RA-peptide]. Therefore, 14 out of 27 previously positive patients with immune-mediated disease became negative using the new O.D. threshold level.

Then, we evaluated the diagnostic power of our test by assessing the sensitivity, specificity, positive predictive power (PPV), negative predictive power (NPV) and accuracy, by using the two different cutoffs (0.079 or 0.090) and by comparing to healthy donors both seronegative and seropositive RA patients (**Tables 2A, B** respectively).

**Table 2:** Association of high level of anti-RA peptide Abs with diagnosis of RA.

**A**		**cut-off = 0.079**				**cut-off = 0.090**		
		**Positive**	**Negative**	**total**		**Positive**	**Negative**	**total**
**RA seronegative patients** (%)		**60** (75%)	**20** (25%)	**80**		**59** (73,8%)	**21** (26,3%)	**80**
**Healthy donors** (%)		**0** (0%)	**25** (100%)	**25**		**0** (0%)	**25** (100%)	**25**
	**total**	**60**	**45**	**105**	**total**	**59**	**46**	**105**
**sensitivity**	**75%**	95% CI: 0.6406 to 0.8401			**73.8%**	95% CI: 0.6271 to 0.8296		
**specificity**	**100%**	95% CI: 0.8628 to 1.000			**100%**	95% CI: 0.8628 to 1.000		
**PPV**	**100%**	95% CI: 0.9404 to 1.000			**100%**	95% CI: 0.9394 to 1.000		
**NPV**	**55.6%**	95% CI: 0.4000 to 0.7036			**54.3%**	95% CI: 0.3901 to 0.6910		
**accuracy**	**81%**				**80%**			
**B**		**cut-off = 0.079**				**cut-off = 0.090**		
		**Positive**	**Negative**	**total**		**Positive**	**Negative**	**total**
**RA seropositive patients** (%)		**36** (65.5%)	**19** (34.5%)	**55**		**35** (63.6%)	**20** (36.4%)	**55**
**Healthy donors** (%)		**0** (0%)	**25** (100%)	**25**		**0** (0%)	**25** (100%)	**25**
	**total**	**36**	**44**	**80**	**total**	**35**	**45**	**80**
**sensitivity**	**65.5%**	95% CI: 0.5142 to 0.7776			**63.6%**	95% CI: 0.4956 to 0.7619		
**specificity**	**100%**	95% CI: 0.8628 to 1.000			**100%**	95% CI: 0.8628 to 1.000		
**PPV**	**100%**	95% CI: 0.9026 to 1.000			**100%**	95% CI: 0.9000 to 1.000		
**NPV**	**56.8%**	95% CI: 0.4103 to 0.7165			**55.6%**	95% CI: 0.4000 to 0.7036		
**accuracy**	**76.3%**				**75%**			

**(A)** RA seronegative patients and healthy donors; **(B)** RA seropositive patients and healthy donors; diagnostic powers of the Elisa test. High levels are defined as above the threshold value of mean +/- 3 S.D. of healthy donors (HD), cut-off = 0.079; or above a hypothetical cutoff value with a more stringent threshold, cut-off = 0.090. Data are analyzed as categorical variable with GraphPad Prism 5. Fisher’s exact test, two-sided; p value ˂ 0.0001. Abs, antibodies; PPV, positive predictive value; NPV, negative predictive value.

From the data obtained by this analysis, we consider the arbitrary cutoff as a valid limit. Therefore, by pooling both cohorts of seronegative RA patients, we observed that 59 out of 80 (73.8%) sera were characterized by the presence of IgG antibodies against the RA-peptide, which were present in 35 out of 55 (63.6%) seropositive RA patients.

### Associations of O.D. With Clinical and Laboratory Parameters and O.D. Quartile Distribution of RA Patients Taking or Not Corticosteroid Therapy

Within the group of seronegative RA patients there was no difference in O.D. values between females and males (0.182 ± 0.131 vs 0.131 ± 0.062, p=0.465) or between subjects with or without joint erosions (0.253 ± 0.183 vs 0.155 ± 0.099, p=0.151). There was no correlation between O.D. levels and age at enrollment, age at diagnosis, disease duration, ESR, CRP, DAS28, tender and swollen joint counts (**Supplementary Table 2A**). There was no difference in O.D. values among patients receiving different disease modifying anti-rheumatic drugs (**Supplementary Table 2B**), while treatment with corticosteroids was associated with lower O.D. levels (0.132 ± 0.095 vs 0.193 ± 0.127, p=0.028 by t-test).

**Figure 2:**
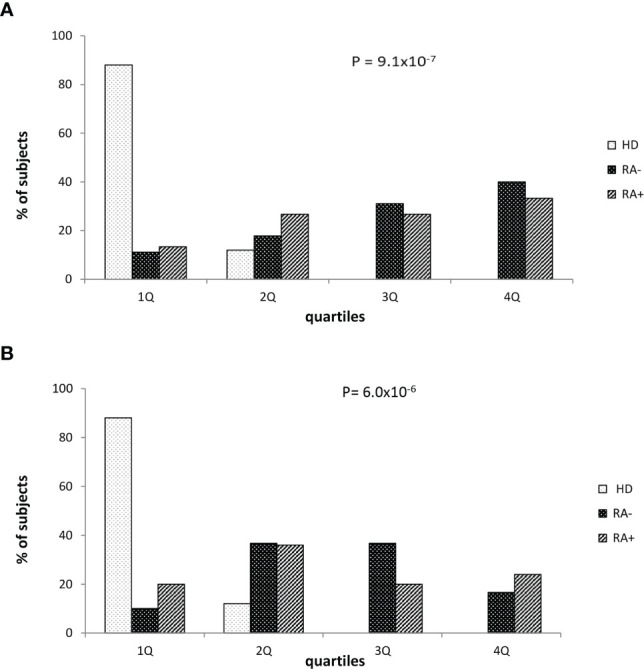
Healthy donors and patients with seronegative or seropositive arthritis according to the quartile distribution of absorbance levels in patients who were not **(A)** or were treated with corticosteroid **(B)**. RA seronegative (RA-) patients, RA seropositive (RA+) patients and healthy donors (HD). Quartile distribution was determined on the basis of the following threshold values: Q1 < 0.045; 0.045 ≤ Q2 ≤ 0.112 or Q2 0.045-0.112; 0.113≤ Q3 ≤ 0.197 or Q3 0.113-0.197; Q4 ≥ 0.198. Statistical p value was obtained by χ^2^ for linear trend.

Finally, we stratified the whole study sample according the O.D. quartile distribution. Considering the potential influence of corticosteroid therapy, these analyses were performed in subgroups of subjects taking or not corticosteroids (**Figure 2**). As expected, healthy donors were strictly prevalent in the lowest quartile while seropositive RA (RA+) or seronegative RA (RA-) patients were more frequent in the higher quartiles in both the subgroups. A progressive increase of prevalence of RA+/RA- patients by increasing O.D. values, from the lowest to the highest quartile, was particularly evident among subjects not taking corticosteroids (**Figure 2A**), while the trend – although significant – appeared milder in those taking corticosteroids (**Figure 2B**).

### Sequence Homology Between the Identified RA-Peptide and Human Proteins

**Figure 3:**
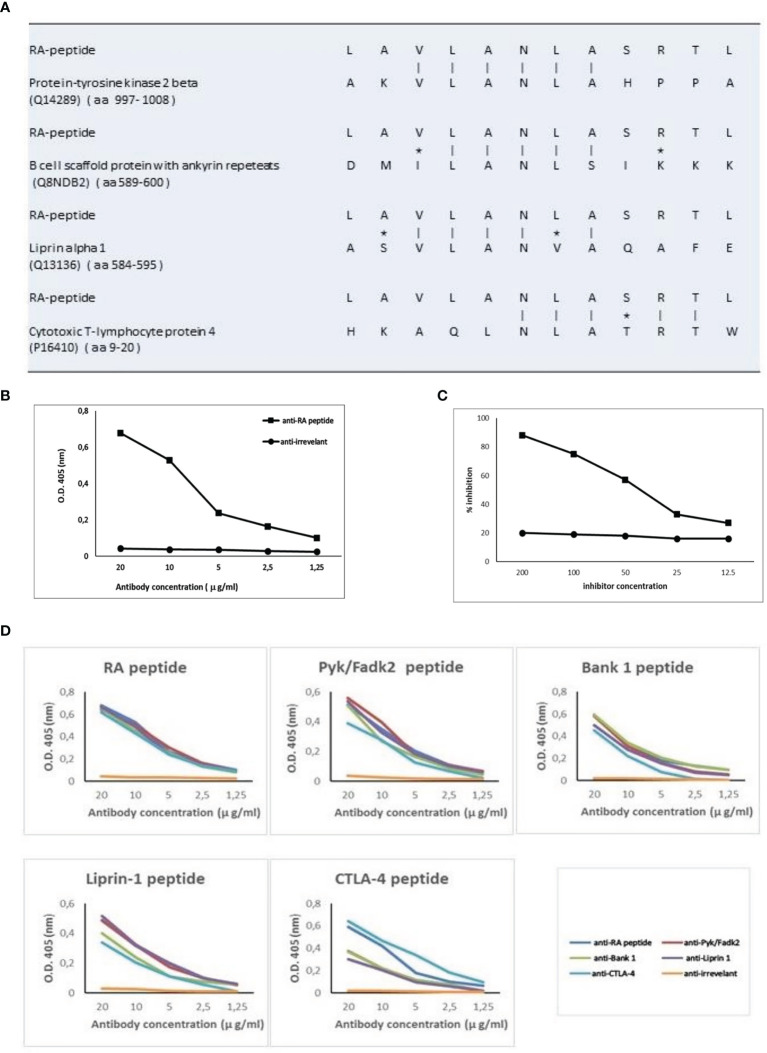
RA-peptide shares sequence homology with human self-proteins and anti-RA-peptide antibodies are cross-reactive. **(A)** Sequence homology between RA-peptide and autoantigens, analyzed by the basic local alignment search tool using the National Center for Biotechnology Information (NCBI) network service. Vertical line = identical amino acids; asterisk= conservative substitutions. **(B)** Direct binding of affinity purified anti-RA-peptide antibodies or anti-irrelevant peptide antibodies to RA synthetic peptide evaluated by ELISA assay. Data are plotted in a graph displaying absorbance (O.D.) at 405 nm on the vertical axis and antibody concentration expressed in μg/ml on horizontal axis. **(C)** Inhibition ELISA test: the binding of affinity-purified antibodies to RA-peptide was inhibited by RA synthetic peptide (■), but not by an irrelevant peptide (●). On vertical axis the inhibition percentage is reported, while on horizontal axis the inhibitor concentration (μg/ml) is shown. **(D)** Specific binding of cross-reactive IgG antibodies to peptides: affinity-purified antibodies against RA (blue line), Pyk/Fadk2 (red line), Bank-1 (green line), Liprin-1 (purple line), CTLA-4 (light blue line) and irrelevant (orange line) peptides were used in a direct binding assay with each one of the 5 synthetic peptides. Graph displays absorbance (O.D.) at 405 nm on vertical axis and antibody concentration μg/ml on horizontal axis.

The 12 amino acid sequence of RA-peptide obtained by the peptide library was compared with known human proteins in a protein data bank (Swiss-Prot database) using BLASTP program *via* the NCBI BLAST network service (https://blast.ncbi.nlm.nih.gov/). In the protein selection, we considered the extent of the homology by measuring the length of the homologous stretch and the number of matched amino acids (both identities and conservative substitutions). Through this approach it is possible to identify areas of homology between the RA-peptide and known human proteins We found that the RA-peptide shares homology with different self-antigens, such as: Protein-tyrosine kinase 2 beta (PYK2/FADK2), B cell scaffold protein with ankyrin repeats (BANK-1), Liprin-alpha 1 (LIPRIN-1), Cytotoxic T-lymphocyte protein 4 (CTLA-4) (**Figure 3A**).

### Direct or Competitive Elisa Assays

Fifteen sera from seronegative RA patients were used to obtain specific IgG antibodies directed against the RA-peptide and against an irrelevant peptide. These affinity-purified anti-RA-peptide Abs were able to bind the RA-peptide in a direct ELISA test (**Figure 3B**), while the anti-irrelevant-peptide Abs did not have this ability. Furthermore, RA-peptide was able to inhibit direct binding of purified anti-RA-peptide Abs. Such inhibition was not present using the irrelevant control peptide (**Figure 3C**).

Antibodies directed against the other peptides were obtained from the same sera of 15 seronegative RA patients (see **Supplementary Material and Methods**). We investigated the ability of these affinity-purified IgG antibodies to bind to their specific peptides (RA, PYK2/FADK2, BANK-1, LIPRIN-1 and CTLA-4 peptide) and to cross-react with the other peptides in ELISA assays, using different concentrations of anti-peptide antibodies (from 20 to 1.25 μg/ml).

All the purified antibodies bind their specific peptide with high affinity and in a dose dependent manner, whereas antibodies against the irrelevant peptide do not show similar behavior (**Figure 3D**).

Anti-CTLA-4 peptide purified antibodies displayed a lower affinity binding to PYK2/FADK2, BANK-1 and LIPRIN-1 peptides, even when we used different concentrations of antibodies. Moreover, anti-CTLA-4 and anti-RA-peptide purified antibodies showed a similar binding to CTLA-4 peptide while the other Abs displayed a lower recognition of this peptide (**Figure 3D**). We supposed that these differences in affinity binding derive from the differences in the sequence homology, as shown in **Figure 3A**.

We check whether the serum samples from the validation cohort were able to cross-react with the other four peptides (arbitrary cutoff = 0.090)

**Figure 4:**
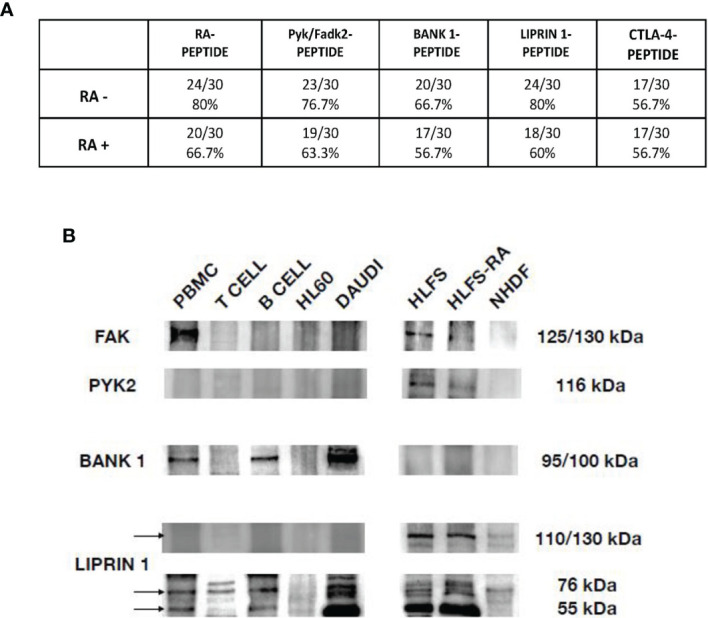
Peptides cross-reaction of RA patients’ sera and recognition of auto-antigens by purified anti-RA-peptide Abs. **(A)** Frequency of the direct binding of seronegative and seropositive patients’ sera to RA, Pyk/Fadk2, Bank-1, Liprin-1 and CTLA-4 peptides evaluated by ELISA assays. No binding was observed using serum IgG of healthy donors. The arbitrary cutoff of 0.090 was used. **(B)** Immunoblot analysis of cell lysates, after immunoprecipitation with affinity purified anti-RA-peptide antibodies detected with commercial antibodies: mouse polyclonal antibodies directed against PYK2 or rabbit polyclonal antibodies directed against BANK, or rabbit polyclonal antibodies directed against Liprin. PBMCs, T or B cells were obtained by a normal healthy donor; HL60 (Human promyelocytic leukemia) and DAUDI (Burkitt lymphoma cell) lines were used as controls; HLFS (Normal Human Fibroblast-Like Synoviocytes) and HLFS-RA (Human Fibroblast-Like Synoviocytes: Rheumatoid Arthritis) and NHDF (Normal human dermal fibroblast) were commercially obtained.

In **Figure 4A** we showed the direct reactivity of RA patients’ sera to PYK2/FADK2, BANK-1, LIPRIN-1 and CTLA-4 peptides. In the cohort of seronegative RA patients, 23 out of 30 patients’ sera (76.7%) had IgG antibodies directed against the peptide PYK2/FADK2, while 20 out of 30 (66.7%) against the BANK-1 peptide. Interestingly, 80% of patients positively reacted with LIPRIN-1, that is the percentage of patients’ sera positive to RA-peptide. No difference between seronegative and seropositive patients (56.7%) was observed in CTLA-4 peptide recognition. In the 30 seropositive patients’ sera (validation cohort), IgG antibodies directed against PYK2/FADK2, BANK-1, LIPRIN-1 peptides were present in 19 (63.3%), 17 (56.7%) and 18 (60%) patients, respectively (**Figure 4A**). None of the 25 healthy donors, used as a control, had IgG antibodies directed against all these peptides.

### Recognition of Auto-Antigens by Anti-RA-Peptide Purified Antibodies

In order to investigate whether the affinity purified antibodies against the RA-peptide can recognize autoantigens, we performed western blot and immunoprecipitation analysis. By the data obtained from the direct binding of purified CTLA-4 Abs to the different peptides (**Figure 3D**) and from the direct binding of patients’ sera (**Figure 4A**), we excluded the CTLA-4 antigen.

First, we considered the protein-tyrosine kinase 2 beta (also known as PKB, PTK, PYK2, FADK2, FAK2, CAKB, CADTK, RAFTK), a non-receptor protein-tyrosine kinase implicated in many different cellular functions. Some authors reported that PYK2 exhibits the same domain organization and structural characteristics of FAK and they share a high degree of sequence similarity (48% amino acid identity and 65% similar at the protein level). However, the two proteins have differences in their C-terminal domains, which are probably implicated in their different regulation (33). For this reason, FAK and PYK2 are defined as a distinct family of non-receptor tyrosine kinases (34).

We identified two bands of approximately 125/150 kDa and 116 kDa corresponding to FAK and PYK2, respectively (**Figure 4B**), as reported also by other investigators (33, 35, 36).

We found the presence of FAK in PBMCs, but not in T or B cells or in HL60 or DAUDI cell lines. We found also a band at 125 kDa in normal human fibroblast-like synoviocytes and RA human fibroblast-like synoviocytes (HLFS and HLFS-RA, respectively), but we did not find it in normal dermal fibroblast (NHDF). These findings have been already reported by other authors (33, 37).

As regards PYK2, we did not find any band at 116 kDa in normal PBMCs, T or B cells (although some investigators reported that PYK2 band was slightly present in PBMCs from healthy donors (38)), while the expression was present in HLFS and HLFS-RA, and was absent in NHDF.

We then considered the BANK-1 protein, a cytoplasmic scaffold protein expressed in B but not in T cells or myeloid cell lines (39). The expected molecular weight of BANK-1 is around 85 kDa, but it may run at 97-105 kDa in SDS-PAGE.

We found a band approximately around 95/100 kDa only in PBMCs, B cells and DAUDI cell line, which was used as positive control. These data have been already described by other authors, who have used DAUDI as positive control (40). In our analysis, no bands were detected in the HLFS, HLFS-RA and in NHDF.

Finally, we evaluated the Liprin-alpha1, a subclass of liprins, which are cytosolic scaffold dimeric proteins, widely expressed and involved in cell motility. Liprin-alpha and beta usually homodimerize, but they can also form heterodimers or large molecular complexes interacting with other proteins, such as LAR (leukocyte common antigen-related) or integrins (41). We identified two bands of 55 kDa and 76 kDa,corresponding to homodimer and heterodimer of liprin-alpha1 protein, and a band between 110-130 kDa, corresponding to a heterodimer of liprin-alpha 1 with an unknown protein.

As shown in **Figure 4B** the homodimeric form was identified in PBMCs, B cells and DAUDI cell line, and it was present in synoviocytes but absent in dermal fibroblasts. The heterodimeric form was reveled in PBMCs, T and B cells, DAUDI cell line, and in HLFS, HLFS-RA and in NHDF. The higher molecular weight band was only present in HLFS, HLFS-RA and weakly detectable in NHDF. The presence of the high molecular weight band in fibroblasts has also been described by other authors (42, 43).

## Discussion

Since the comprehension of pathogenesis of autoimmune diseases is crucial both for early diagnosis and treatment, the identification of novel autoantigens may play fundamental role.

The diagnosis of seropositive RA is based on the detection of serological markers such as rheumatoid factor (RF) and anti-citrullinated peptide antibodies (ACPAs) (15), which may be present years before the onset of the disease (2). ACPAs can be also detected in the serum of subjects with undifferentiated arthritis, who later on will develop RA (44, 45). On the other hand, seronegative RA does not have the diagnostic help of any laboratory biomarkers so far and its definition still remains a clinical challenge.

The random peptide library is a well-known and useful molecular biology tool for the identification of possible immunodominant peptides in autoimmune disorders (26–28). The screening of peptide library with pooled immunoglobulins from patients affected by an autoimmune disease leads firstly to the identification of immunodominant peptides acting as autoantigens. Secondly, it leads to the identification of antibodies able to bind such peptides and autoantigens, in the patients’ sera. Therefore, the presence of these auto-antibodies in patients’ sera may be helpful in the diagnosis of the disease and, if they are functionally active, they may provide new relevant information on the pathogenesis of the studied autoimmune disease (26–28).

In the present work, by using this approach in seronegative RA patients, we could identify a peptide, named RA-peptide, which is specifically recognized by both seronegative and seropositive RA patients’ sera (70.0% and 64.0%, respectively) but not by any sera from healthy donors. Such findings were confirmed in a second validation cohort of RA patients with similar results (83.3% and 66.7%, respectively).

In a second step, we verify whether the recognition of RA-peptide is truly specific for RA. To achieve this aim, we enrolled three other cohorts of patients affected by different immune-mediated diseases. We observed that, in these three groups many patients’ sera show an absorbance value close to the standard cutoff (means ± 3 s.d. of the healthy control group). The ROC curve and contingency table analyses induced us to apply a second arbitrary cutoff (0.090). Using this cutoff, we observed that 73.8% and 63.3% of all seronegative or seropositive RA patients’ sera, respectively, recognised the RA-peptide, while a greater number of patients with other immune-mediated diseases resulted negative.These data indicate that this RA-peptide sequence is recognized by the sera of patients affected by RA disease at high frequency, independent of RF and ACPAs positivity, but not by the sera obtained from healthy donors or from patients with other immune-mediated diseases, thereby suggesting that antibodies directed against this epitope are highly specific for RA.

Within the group of seronegative RA patients, we found no correlation of anti-RA-peptide Abs with disease activity or inflammatory markers. This apparent discrepancy may be consistent with the findings in the different context of seropositive RA, where some authors observed that many patients treated with disease-modifying agents showed a decrease in inflammation and pain but not in ACPA levels (46).

As regards corticosteroid therapy, by analysing the quartile distribution of anti-RA-peptide antibody levels among our RA patients, we observed that patients on corticosteroid therapy were mostly in the second and/or third quartiles, while those never treated with steroid were mostly in the fourth quartile. This observation suggests that corticosteroid therapy may lead to a reduction in the levels of anti-RA-peptide Abs in RA patients’ sera. Interestingly, in other autoimmune diseases we showed that corticosteroid therapy lowers the levels of Abs specifically in good responder patients (47).

However, even if corticosteroid therapy may lower Abs levels, it is worthy to note that such level remains higher than in healthy subjects, thus confirming that anti-RA-peptide Abs may be a biomarker for RA.

As above mentioned, RA-peptide shares sequence homology with proteins expressed by cells that are considered to be important in RA, such as Protein-tyrosine kinase 2 beta (PYK2/FADK2), B cell scaffold protein (BANK-1), Liprin-alpha 1 (LIPRIN-1), and Cytotoxic T-lymphocyte protein 4 (CTLA-4). For this reason, we wanted to evaluate whether affinity purified anti-RA-peptide Abs were also able to recognize these four proteins. To this aim, we selected four peptides (one of each protein) based on the sequence homology shared with RA-peptide. We then purified Abs from patients’ sera directed against each one of these peptides and test their ability to cross react. We observed that these affinity-purified Abs, not only were able to recognize the peptide used for the purification, but showed a dose-dependent cross reactivity with all the other peptides. On the contrary, the Abs purified against the irrelevant peptide were not able to bind any of the five peptides, even at high concentration. We also confirm that the sera of RA patients can positively react with all the peptides.

Notably, the identified four proteins are present in cells involved in RA pathogenesis, such as B and T lymphocytes, macrophage/monocytes, fibroblast-like synoviocytes, fibroblasts, osteoclasts and endothelial cells, thereby being consistent with a potential biological role in this immune-mediated disease.

Several studies on RA have described differences in B cell subsets distribution in both peripheral blood and bone marrow before and after B cell depletion treatments (48). No differences were found at peripheral blood level between healthy donors and RA patients. Moreover, B cell subpopulations showed the same distribution among seronegative-, seropositive- and non-RA patients both in peripheral blood and synovial fluid (49). A study by Michelutti et al. showed that specific B cell subsets are present in the synovium of both seronegative and seropositive RA patients. Moreover, a recruitment of immature B cells into synovial fluid from peripheral blood was observed and, according to authors’ hypothesis, a persistent inflammation may promote a B cell maturation directly in the joints (50).

Fibroblasts and related different subpopulations play a role in RA pathophysiology. Fibroblast-like synoviocytes (FLS) are aggressively proliferating in the joint synovium of RA patients and such proliferation leads to the invasion and destruction of adjacent cartilage (51, 52). Moreover, the inflammatory environment present in rheumatoid joints may trigger the proliferation of synovial fibroblasts (RA-SF) leading to an increase of the synovial pannus (53) with invasion of healthy cartilage (54, 55). We confirmed that the anti-RA-peptide Abs were able to recognize the three auto-antigens PYK2/FADK2, BANK-1 and LIPRIN-1, which are expressed in the above mentioned cell lines and may be involved in RA pathogenesis. We found that the anti-RA-peptide Abs are able to bind PYK2 and FAK proteins in PBMCs, HLFS, HLFS-RA cells (fibroblast-like synoviocytes).

PYK2 and FAK form a unique group of non-receptor tyrosine kinases. The expression of protein-tyrosine kinase 2 beta (Pyk2) is limited to epithelial cells, neurons, osteoclasts and hematopoietic cells (56, 57), while the focal adhesion kinase (FAK) is expressed in all tissues and, interestingly, in fibroblasts or synovial fibroblasts (37).

*In vitro* it has been shown that FAK plays a central role in arthritic synovial fibroblast invasion and cytokines production. In FAK-knockout mice, the FAK depletion does not reduce murine inflammatory arthritis (37). The authors suggest that FAK’s homologue, Pyk2, may counterbalance the absence of FAK in this mice model. Phosphorylation of Pyk2 can be induced by TNF-α in rheumatoid synovial fibroblasts and in rheumatoid synovial tissue, a large amount of phosphorylated Pyk2 has been found. For this reason, it is likely that these two homologous proteins may equally contribute to inflammatory arthritis. It has been shown that FAK is crucial for synovial fibroblast invasion while it is not for TNF-α-driven erosive arthritis. Because of the homology with FAK and because of Pyk2 phosphorylation is induced by TNF-α, Pyk2 may play a role in erosive arthritis. Even if further studies are needed to better clarify the role of Pyk2 in inflammatory arthritis, it is likely that both proteins are involved in the pathophysiology of RA (37).

We found that anti-RA-peptide Abs can bind Bank-1 protein in PBMCs, B cells and DAUDI cell line. Bank-1 is involved in the regulation of B cell responses, such as CD40-mediated Akt activation and can prevent an excessive B cell activation (39). It is associated with increased anti-nuclear Abs levels (58) and with many different autoimmune diseases, such as RA (59). In collagen-induced arthritis in mice, a decreased expression of Bank-1 facilitates B cell response, increasing antigen presentation and leading to the production of autoantibodies (60).

Finally, anti-RA-peptide Abs can recognize both the homodimeric and heterodimeric forms but also large molecular complexes of Liprin-alpha 1 protein in the extract of different cell types. The higher molecular weight band was identified in fibroblast-like synoviocytes (HLFS, HLFS-RA) and to a lower extent in dermal fibroblasts (NHDF), but it is not present in PBMCs, B, T, HL60 and DAUDI cells. Evident bands (homodimeric and heterodimeric forms) could be observed in DAUDI cells, a tumor B cell line, and in fibroblast-like synoviocytes. Liprin-alpha 1, as other members of the liprin family proteins, is involved in forming and maintaining the cellular cytosolic scaffold (41, 61, 62) and in the formation of adhesion foci. Notably, it is present in the cytoskeletal core of podosomes, essential elements for cell motility (63–65).

This study had some strength and limitations, which should be acknowledged. The random peptide library is a method that allows the screening of a huge number of different 12 amino acid peptides in a faster and easier way than a phage library. Those peptides are obtained by a random sequence in the flagellin of cultured E. Coli. When this method is used to search for unknown autoantibodies the criteria driving the choice of the patients’ sera to be used are crucial. The use of healthy donors’ sera as a prescreening step helps to eliminate from subsequent analysis those peptides that have shown some reactivity, although it may potentially lead to loss of data. Nonetheless, this can save time and decrease the number of screening steps required to finally recognize new etiopathological relevant epitopes. Our results show that RA peptide is recognized by the sera of seronegative RA patients. Speculatively, an *ad hoc* ELISA test can improve the diagnosis of this disease. However, more studies need to be done before such ELISA test could be considered as a validated tool. Larger cohorts of patients, possibly including different ethnicity, need to be investigated to check the specificity and accuracy of the test.

## Conclusion

This study investigates potential candidate biomarkers of seronegative RA by peptide library screening. Here we show that RA-peptide is recognized by antibodies present in the sera of RA patients, particularly in the seronegative subjects.

RA-peptide shows similarity with four epitopes contained in four different proteins which may be involved in RA pathogenesis such as PYK2/FADK2, BANK-1, LIPRIN-1, and CTLA-4.

The anti-RA-peptide Abs are able to bind both the homologous epitopes and the full proteins. Therefore, we can consider the new identified autoantibodies as a specific biomarker of seronegative RA and RA-peptide could be used as a tool for the development of diagnostic assays for seronegative RA.

## Data Availability Statement

The raw data supporting the conclusions of this article will be made available by the authors, without undue reservation.

## Ethics Statement 

The study was approved by the local Ethical Committee of the Azienda Ospedaliera Universitaria Integrata of Verona, Verona, Italy (protocol number: 1538, version number 3) and by Comitato Universitario di Bioetica of Perugia, Italy (identification code: 2013-012 approved 4-4-2013). All clinical investigations were conducted according to the principles expressed in the Helsinki declaration. The patients/participants provided their written informed consent to participate in this study.

## Author Contributions

CB conceived and designed the study, performed the experiments, collected, analyzed and interpreted data and wrote the manuscript. AB provided useful suggestions and wrote the manuscript. NM did the statistical analysis. BO, EB, ET, and AP recruited patients and collected the clinical and demographic data. GA, RB and GJ collected the samples and provided reagents. ET and CL provided funding acquisition. CL conceived the study, supervised the work, wrote and gave the final approval of the manuscript. All authors contributed to the article and approved the submitted version.

## Funding

This research was partially supported by a grant from the University of Verona (CL) and by FIRB (Futuro in Ricerca-Bando Giovani) 2010, project number RBFR10A0G1 (ET).

## Conflict of Interest

The authors declare that the research was conducted in the absence of any commercial or financial relationships that could be construed as a potential conflict of interest.

## Publisher’s Note

All claims expressed in this article are solely those of the authors and do not necessarily represent those of their affiliated organizations, or those of the publisher, the editors and the reviewers. Any product that may be evaluated in this article, or claim that may be made by its manufacturer, is not guaranteed or endorsed by the publisher.

## Abbreviations

Abs, antibodies; ACPAs, anti-citrullinated peptide antibodies; AUC, area under the curve; BANK-1, B cell scaffold protein with ANKyrin repeats; BSA, bovine serum albumin; CI, confidence interval; CRP, C reactive protein; CTLA-4, cytotoxic T lymphocyte protein 4; DAS 28, disease activity score 28; DAUDI, Burkitt lymphoma cell; ELISA, enzyme-linked immunosorbent assay; ESR, erythrocyte sedimentation rate; HD, healthy donors; HFLS, normal human fibroblast-like synoviocytes; HFLS-RA, human fibroblast-like synoviocytes: rheumatoid arthritis; HL-60, human promyelocytic leukemia cell; IgG, immunoglobulin G; IP, immunoprecipitation; LIPRIN-1/LIP-1, liprin-alpha 1; mAbs, monoclonal antibodies; NHDF, normal human dermal fibroblast; O.D., optical density; PBMCs, peripheral blood mononuclear cells; PBS, phosphate-buffered saline; PsA, psoriatic arthritis; PYK2/FADK2, protein-tyrosine kinase 2 beta/focal adhesion kinase; RA, rheumatoid arthritis; RF, rheumatoid factor; ROC, receiver operating characteristic; RPLs, random peptide libraries; SA, spondyloarthritis; S.D., standard deviation; SF, synovial fluid; SSc, systemic sclerosis; SDS-PAGE, sodium dodecyl sulphate-polyacrylamide gel electrophoresis; SJ, swollen joint count; TBS-T, tris-buffered saline containing 0.1% Tween 20; TJ, tender joint count; WB, western blot.
