# Supplementary material for: Identification of a Novel Serological Marker in Seronegative Rheumatoid Arthritis Using the Peptide Library Approach
Source: Front Immunol. 2021 Oct 5;12:753400. doi: 10.3389/fimmu.2021.753400 (PMC8525329; doi:10.3389/fimmu.2021.753400)
Supplement: Supplementary file 1 [file DataSheet_1.pdf]

Supplementary material and methods

Enzyme-linked immunosorbent assays (ELISA)

Briefly, the synthetic peptides were used at a concentration of 20 µg/mL in phosphate-buffered saline (PBS) pH 7.4 to coat polystyrene plates (Immulon II, Dynax, Ashford, UK). The plates (half coated and half not coated with peptides) were then blocked for 1 h with PBS 3 % BSA. Serum samples were diluted 1:100 in diluting buffer (PBS 1 % BSA) and incubated overnight at 4° C. The washing steps were performed once with Tween at 1% in PBS and twice with PBS alone. Alkaline phosphatase-conjugate anti-human IgG antiserum (Sigma, St. Louis, MO, USA) was added and incubation lasted for 3 hours at room temperature. After washing, alkaline phosphatase substrate (Sigma) was added and the plates were evaluated after 30 min or 1 hour using a microplate absorbance reader (SunriseTM III, Tecan, Männedorf, CH) set at 405 nm.

Affinity Purification of Anti-peptide Antibodies

The 5 peptides (5 mg peptide per gram of dried Sepharose powder) were coupled to Sepharose 4B (Pharmacia Biotech, Piscataway, New Jersey) following manufacturer's instructions. Sera from 15 seronegative RA patients were diluted in PBS and applied to the different columns. IgGs bound to the specific peptides were eluted with 0.1 mol L-1 glycine (pH 2.5) and dialyzed against PBS. The purity of the preparations was confirmed by a SDS-PAGE followed by silver staining. The concentration of affinity purified IgGs was determined by Pierce BCA Protein Assay Kit (Thermo Scientific, Rockford, IL).

Supplementary Table 1. Clinical and laboratory features and treatment of seropositive rheumatoid arthritis (RA) patients from the Perugia (Pg) and Verona (Vr) cohorts.

Clinical characteristics	**RA (Pg)**<br>**(n=25)**	**RA (Vr)**<br>**(n=30)**
**Age (years)**	55.88 ± 12.15	58.9 ± 11.45
**Gender: female/male, n.**	18/7	25/5
**Diagnosis time (years)**	53.48 ± 13.38	46.4 ± 11.05
**Disease duration (years)**	2.4 ± 5.12	12.5 ± 11.24

Data are shown as mean ± SD

Therapy	**RA**<br>**(n=25)**	**RA**<br>**(n=30)**
**Methotrexate (%)**	19 (76%)	17 (56.7%)
**Hydroxychloroquine (%)**	3 (12%)	1 (3.33%)
**Leflunomide (%)**	1 (4%)	1 (3.33%)
**Biological/others (%)**	2 (8%)	6 (20%)
**no treatment (%)**	0 (0%)	5 (16.7%)
**Prednisolon/prednisone (%)**	4 (16%)	21 (70%)

RA,

rheumatoid arthritis; DAS 28, Disease activity score 28; ESR, Erythrocyte sedimentation rate; CRP, C reactive protein. Values are shown as mean ± SD and as percentage (%).

Supplementary Figure 1. Receiver operating characteristic (ROC) analysis of the patients enrolled in the study.
ROC curves obtained using seropositive RA serum samples. (A) comparison between the seropositive RA and healthy donors group; (B) comparison between seropositive and seronegative RA patients.
ROC curves obtained by seropositive RA serum samples of the validation cohort. (C) sensitivity and specificity of anti-RA-peptide antibodies between seropositive RA patients and seronegative RA patients; (D) seropositive RA against SSc sera; (E) seropositive RA against SA; (F) seropositive RA against PsA.
ROC curves were constructed by plotting sensitivity against 1-specificity. AUC and 95% confidence intervals (CI) are shown in each graph. P values are also reported in all graphs.

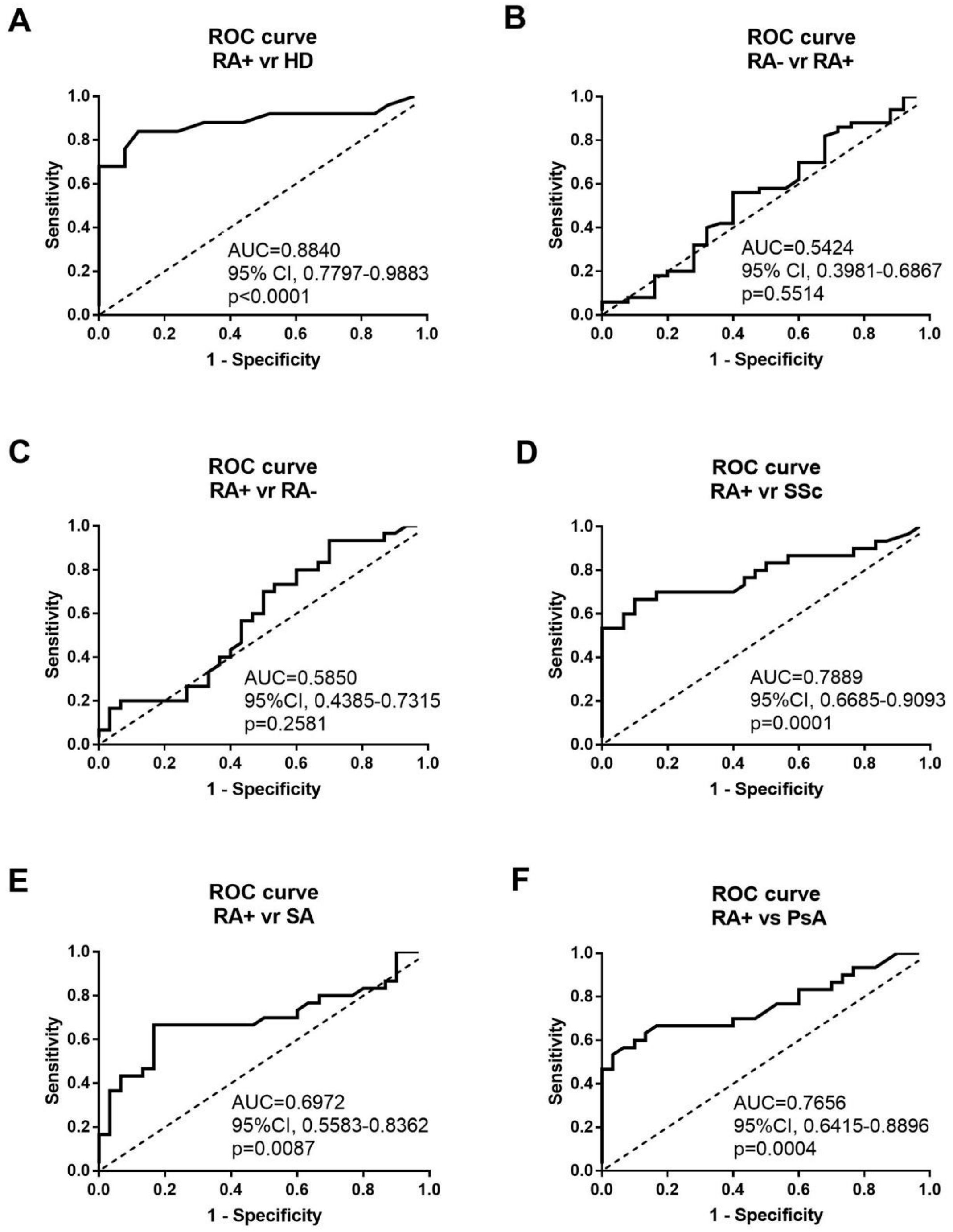

Supplementary Table 2. Correlation analysis between O.D. levels of seronegative RA patients and clinical / laboratory data (A) and difference in O.D. values in patients treated with disease modifying anti-rheumatic drugs (B).

A	Pearson's Correlation			O.D.			
			r value		p value		
	Age (years)		-0.086		0.449		
	Diagnosis time		-0.023		0.842		
	Disease duration		-0.162		0.150		
	Erosion		0.169		0.151		
	Tender joint count		0.008		0.948		
	Swollen joint count		0.095		0.423		
	DAS28-ESR		0.056		0.620		
	ESR		0.107		0.352		
	CRP		0.069		0.550		
B			means ± s.d.			p value	
	Methotrexate		0.179 ± 0.135				
	Hydroxychloroquine		0.158 ± 0.108			0.884	
	Leflunomide		0.139 ± 0.068				
